# A large type I right pulmonary artery to left atrium fistula: underwent successful percutaneous device closure with duct occluder—a rare case report

**DOI:** 10.1186/s43044-024-00438-w

**Published:** 2024-02-21

**Authors:** S. P. Vinothkumar, Satya Sahitya Mandava, Abhishek Mallick, Manphool Singhal, Manoj Kumar Rohit

**Affiliations:** 1 Department of Cardiology, Level-3, Faculty Offices, Advanced Cardiac Centre, Postgraduate Institute of Medical Education and Research, Sector-12, Chandigarh, 160012 India; 2 Department of Radiodiagnosis and Imaging, Postgraduate Institute of Medical Education and Research, Sector-12, Chandigarh, 160012 India

**Keywords:** Cyanosis, RPA-LA fistula, Device closure, Case report

## Abstract

**Background:**

Pulmonary artery to left atrium fistula is an unusual structural cause of silent cyanosis. Only less than 100 cases have been reported so far. A high index of clinical suspicion and proper evaluation with bubble contrast echocardiography and cardiac computed tomography (CT) will help to detect this treatable anomaly. The advent of safer percutaneous closure methods has replaced the need for more invasive surgical closure.

**Case presentation:**

We report an adolescent boy, who presented with long-standing cyanosis and progressive dyspnea with normal clinical cardiovascular examination. On evaluation, echocardiography and bubble contrast study revealed a large right pulmonary artery (RPA) to left atrium (LA) fistula. Cardiac CT confirmed the same with normal pulmonary venous drainage s/o a large 20 mm Type I RPA LA Fistula. He underwent successful percutaneous closure of the fistula tract with a 22 × 24 mm Cera™ duct occluder via transseptal approach uneventfully.

**Conclusion:**

Our case enlightens the methodological approach to diagnosing this rare anomaly as well as the feasibility of percutaneous intervention in such cases as it is one of the largest fistula tracts closed percutaneously to date.

**Supplementary Information:**

The online version contains supplementary material available at 10.1186/s43044-024-00438-w.

## Background

The pulmonary artery-to-left atrium fistula is a rare cause of cyanosis. Though the initial description was made by Friedlich et al. in 1950, less than 100 cases have been reported so far [1]. We report a large right pulmonary artery (RPA) to left atrium (LA) fistula diagnosed by bubble contrast study aided transthoracic echo and cardiac computed tomography (CT). He underwent successful transseptal percutaneous device closure with a large duct occluder without any complications.

## Case presentation

A sixteen-year-old boy was referred to our institute for the evaluation of cyanosis. Though the parents had noticed cyanosis since early infancy, unfortunately, he was not evaluated earlier. Because of the development of progressive exercise intolerance, the child was referred for further evaluation. There was no history of recurrent chest infections or cyanotic spells. He was otherwise thriving well, with a weight of 45 kg and a height of 160 cm.

On examination, he had pan-digital grade 3 clubbing, cyanosis, and a room air saturation of 63%. His cardiovascular examination revealed normal first and second heart sounds with a normal split and no murmurs. His electrocardiogram and chest x-ray were also unremarkable. His hemoglobin was 22 g/dl with a hematocrit of 66%. Considering the unusual presentation of long-standing silent cyanosis, anomalous systemic venous connections and pulmonary arteriovenous malformations were kept as possible differentials after clinical examination.

**Fig. 1:**
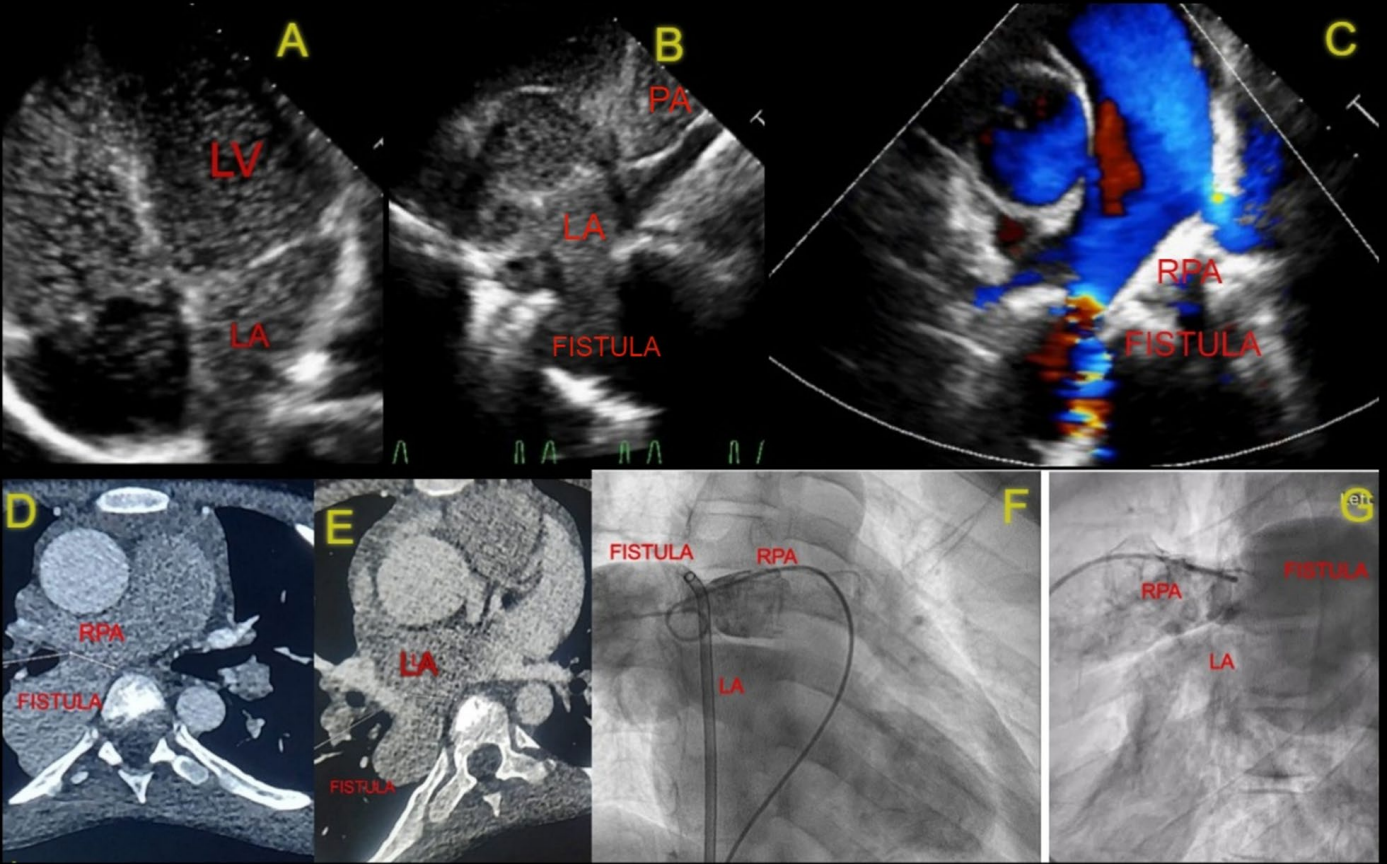
Imaging of the RPA-LA fistula: echo images showing positive bubble contrast study—apical four chamber view showing bubbles filling the left-sided chambers after 3rd cardiac cycle (**A**), parasternal short axis view showing bubbles entering the LA from the fistula tract (**B**) and the colour doppler imaging of the fistula tract between RPA and LA (**C**)*.* CT angio-coronal section images showing RPA LA fistula with aneurysmal sac (**D**, **E**). Angiographic images showing the RPA LA fistula in antero-posterior and lateral view (**F**, **G**) (*LA* left atrium, *LV* left ventricle, *RPA* right pulmonary artery)

His echocardiography showed normal single-sided superior and inferior vena cava and coronary sinus draining into the right atrium (RA), normal pulmonary venous drainage to the left atrium, patent foramen ovale (PFO) shunting left to right, and a note made of a suspicious communication channel from the proximal RPA to LA with continuous flow. The bubble contrast echo revealed a positive study; the apical four-chamber view showed the filling of the left-sided chambers after the 3rd cardiac cycle, and the parasternal short axis view also confirmed the bubbles filling the LA from RPA through the fistula tract (Fig. 1A,B,C). Contrast-enhanced computed tomography (CT) revealed a Type I RPA LA fistula with normal pulmonary venous drainage (Fig. 1D, E). Hence, we planned to close it percutaneously.

## Procedure

**Fig. 2:**
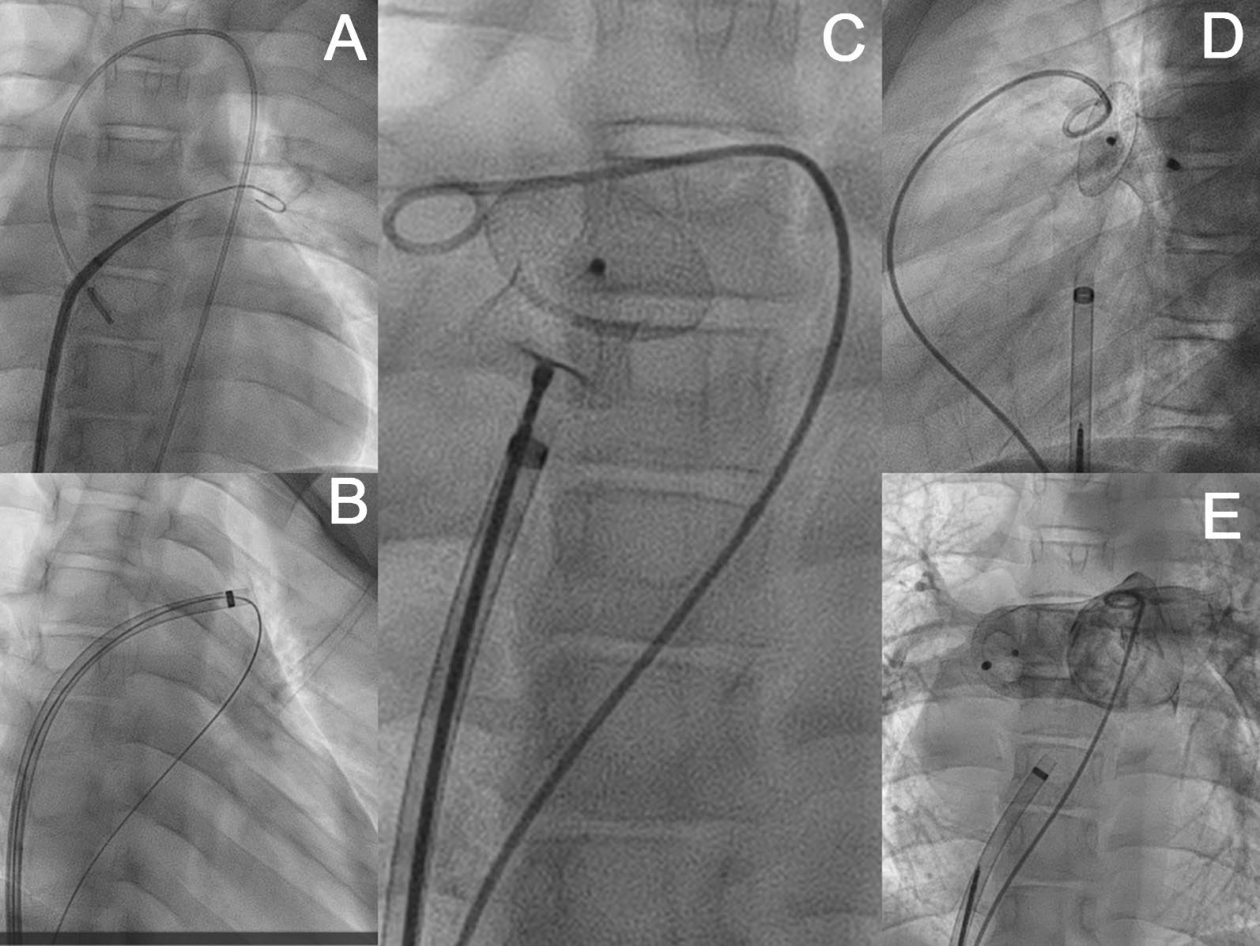
Percutaneous device closure of RPA LA fistula: crossing the PFO with the SL1 sheath and angled tip guide wire (**A**). Formation of veno-venous loop RFV → IVC → RA → LA → Fistula → RPA → MPA → RV → RA → IVC → LFV (**B**). Successful deployment of a 22 × 24 mm Cera™ PDA Occluder (Lifetech Scientific Co. Ltd., Shenzhen, China) (**C**, **D**). Post-release angiographic image showing no residual shunt with good RPA flow (**E**)

After obtaining informed written consent, the child was scheduled for percutaneous intervention. Under intravenous sedation and local anaesthesia, right and left femoral venous access were obtained. A pulmonary artery angiogram performed in lateral and right anterior oblique views showed a fistula tract with an aneurysmal sac between the RPA and LA, with the narrowest opening at the RPA end of 20 mm (Fig. 1F, G) (Additional file 1). Through the right femoral venous route, the PFO was crossed with a multipurpose catheter and an angled tip 0.035-inch guide wire. It was exchanged for Swartz braided transseptal guiding introducer (SL1) sheath, which steered it to enter the fistula sac. An exchange-length guide wire was introduced via left femoral venous access from the main pulmonary artery to the fistula tract. The exchange-length guide wire was snared out through the SL1 sheath to form the veno-venous loop (left femoral vein-RPA-fistula-LA-RA-right femoral vein). A 12-French delivery sheath was advanced over the veno-venous loop from the right femoral vein and traversed through the transseptal route and into the fistulous tract from LA to RPA. A 22 × 24 mm Cera™ duct Occluder (Lifetech Scientific Co. Ltd., Shenzhen, China) was deployed successfully on the floor of the RPA and fistula. A selective RPA angiogram showed a good position of the device with no residual flow across the fistula and good flow to the distal RPA (Fig. 2) (Additional file 2). His saturation improved from 63 to 96% immediately after the closure of the fistula. He was observed for 48 h and discharged without any post-procedural complications. He was doing well on the 6-month follow-up.

## Discussion

**Fig. 3:**
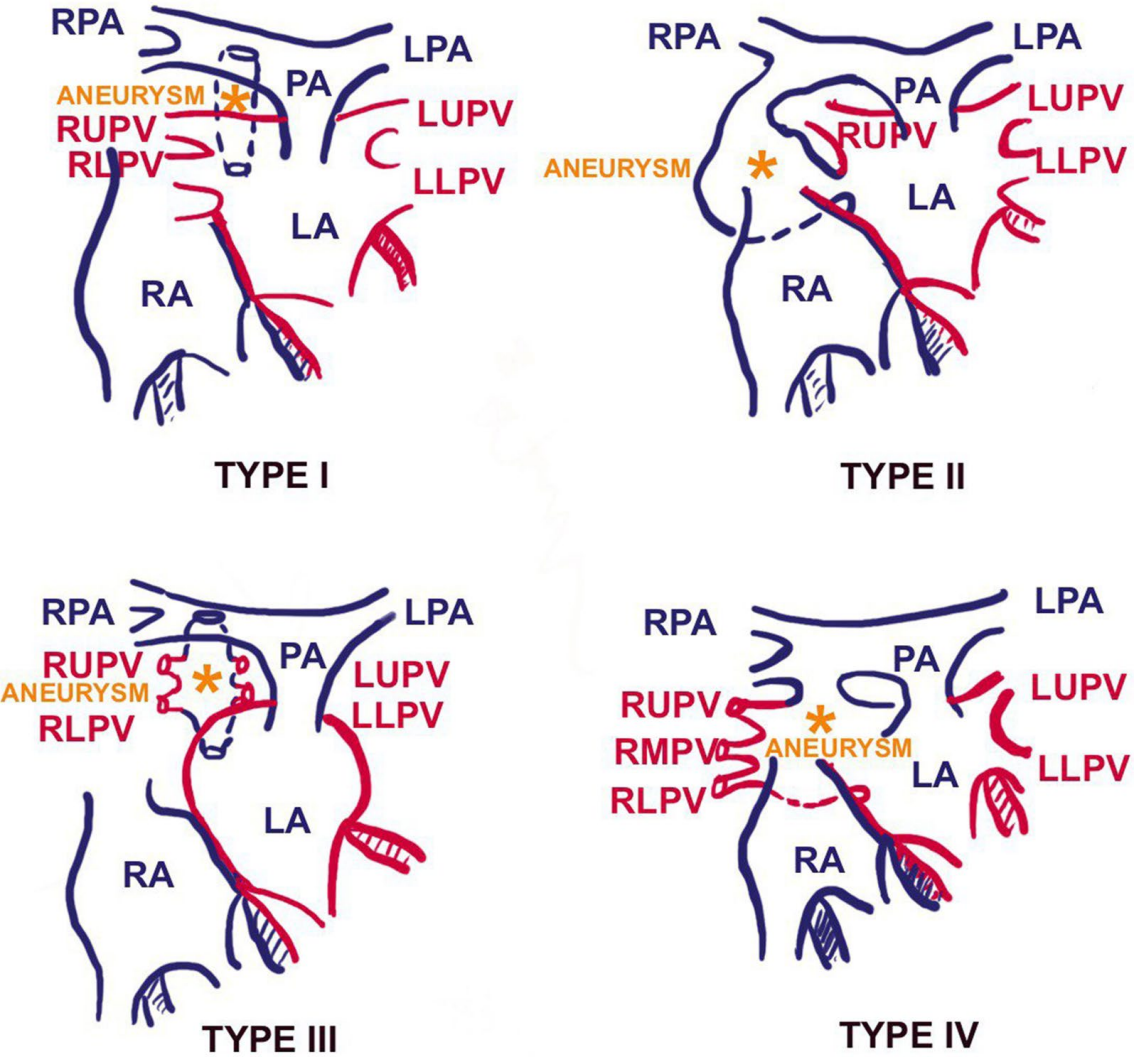
Types of RPA-LA fistula—schematic representation: Type I: proximal RPA-LA fistula with normal pulmonary venous drainage. Type II: Lower lobe branch of RPA forms an aneurysmal sac and connects to LA in the absence of the right inferior pulmonary vein. Type III: The aneurysmal fistula tract receives all the pulmonary venous drainage. Type IV: All right-sided pulmonary veins drain into the aneurysmal sac, with normal left-sided pulmonary venous drainage to LA (*LA* left atrium, *LLPV* left lower pulmonary vein, *LPA* left pulmonary artery, *LUPV* left upper pulmonary vein, *PA* pulmonary artery, *RA* right atrium, *RLPV* right lower pulmonary vein, *RMPV* right middle pulmonary vein, *RUPV* right upper pulmonary vein)

RPA-LA fistula is one of the rare congenital anomalies causing silent cyanosis. There are four types of RPA-LA fistulas described. Type I: RPA branches normally, with an additional fistulous channel connecting RPA and LA. Pulmonary venous return is normal. Type II: The lower lobe branch of the RPA drains directly into the LA, forming an aneurysmal sac in the absence of the right lower pulmonary vein. Type III: All right- and left-sided pulmonary veins drain into the abnormal channel that connects RPA and LA. Type IV: Right-sided pulmonary veins entering the aneurysmal sac of the RPA LA fistula with normal left-sided pulmonary venous drainage to the left atrium (Fig. 3) [2, 3].

In an otherwise structurally normal heart, the bubble contrast echo gives a clue to detect this right-to-left shunt. CT and cardiac catheterization studies will be helpful for confirmation. In our case, the echocardiogram and bubble contrast study itself well delineated the additional fistula tract from the proximal RPA to LA with normal pulmonary venous drainage, suggesting a type I RPA-LA fistula. Cardiac CT and Catheter angiography were used for confirmation and to measure the tract opening precisely for the selection of the appropriate device.

Surgical repair was the preferred mode of management until the first transcatheter coil closure of the RPA-LA fistula was reported by Slack et al. in a sick neonate [4]. Further modifications by using various devices like duct occluders, septal closure devices, and vascular plugs were reported. Francis et al. used a 12 × 14 Amplatzer duct occluder in a 12-year-old child through the atrial septal defect after forming a veno-venous loop, as in our case [5]. Vadlamudi et al. reported a similar case successfully closed with an 18 × 20 duct occluder after a transseptal puncture [6]. Our case might be the one that used the largest duct occluder based on the available literature search to date. Kumar et al. reported a 28-year-old adult patient with an RPA-LA fistula tract closed with a vascular plug [7]. Antegrade deployment of double-disc devices from the RPA to the fistula is an alternate option. However, there is a high risk of RPA occlusion in the pediatric population if it is not deployed properly via the antegrade approach, which is comparatively less common in the transseptal approach. Closure of an RPA-LA fistula with a muscular septal occluder device via an antegrade approach in an adult patient was reported by Ding et al. [8].

Location, size, and tortuosity are important determinants of amenability for device closure. Precise measurement and appropriate device selection are important. Duct occluders, muscular septal closure devices, and vascular plug devices have been used for percutaneous closure. A transseptal approach with a veno-venous loop is the preferred route for device deployment in the pediatric population, especially with a single disc duct occluder. Antegrade approach: closing the fistula from the venous end without looping (Right ventricle → main pulmonary artery → RPA → Fistula) is an alternative, but a double disc device should be used; however, the improper position may compromise distal RPA flow.

## Conclusion

Pulmonary artery to left atrium fistula is an unusual structural cause of silent cyanosis that can almost always be safely treated percutaneously, preferably by transseptal approach.

### Supplementary Information

**Additional file 1: **RPA angiogram showing proximal RPA LA fistula with aneurysmal dilatation and normal distal lower RPA branching.

**Additional file 2: **Post device deployment pulmonary artery angiogram showing good position of the device with no residual shunt, good antegrade flow across RPA and normal diaphragmatic movement.

## Data Availability

All data and materials pertaining to the index case are included in this published article.

## Abbreviations

RPA: Right pulmonary artery
LA: Left atrium
CT: Computed tomography
PFO: Patent foramen ovale
